# Preliminary Investigation of Food Guarding Behavior in Shelter Dogs in the United States

**DOI:** 10.3390/ani2030331

**Published:** 2012-08-03

**Authors:** Heather Mohan-Gibbons, Emily Weiss, Maragret Slater

**Affiliations:** 1 Shelter Research and Development, Community Outreach, American Society for the Prevention of Cruelty to Animals (ASPCA^®^), Ojai, CA 93024, USA; E-Mail: heather.mohan-gibbons@aspca.org; 2 Shelter Research and Development, Community Outreach, American Society for the Prevention of Cruelty to Animals (ASPCA^®^), 6260 N. Hillside, Wichita, KS 67219, USA; 3 Shelter Research and Development, Community Outreach, American Society for the Prevention of Cruelty to Animals (ASPCA^®^), 50 Stone Ridge Drive, Northampton, MA 01602, USA; E-Mail: margaret.slater@aspca.org

**Keywords:** food, resource, possession, aggression, shelter, guarding, adoption, ASPCA

## Abstract

**Simple Summary:**

Even though food guarding is an adaptive trait for dogs, they are often euthanized when they exhibit this behavior while at an animal shelter. This research demonstrates some dogs that guard their food can be adopted and guarding is seldom seen in the home. Based on post-adoption follow-up of the dogs selected for the program, guarding behavior was rarely reported during the first three weeks, and by three months, adopters reported no food bowl guarding behavior. The adopters reported being highly bonded with these dogs and return rates were lower than general shelter dog population. Placing food guarding dogs into homes and providing follow-up support for adopters can provide a life-saving safety net for many shelters.

**Abstract:**

A survey given to animal shelters across the US reported food bowl guarding as one of the most common reasons for euthanasia and only 34% attempted to modify this guarding behavior. This study identified 96 dogs that guarded their food bowl during an assessment, and then placed them into a home on a modification program. Food guarding behavior was identified as stiffening, gulping, growling, freezing, and/or biting a fake hand during the SAFER^®^ food bowl assessment. Dogs that exhibited guarding behavior over toys were excluded. Follow-up was done at 3 days, 3 weeks, and 3 months post adoption to measure all guarding behavior in the home. Six adopters reported at least one incident involving guarding in the first three weeks, of which only one was around the food bowl. By three months, those adopters reported no guarding behavior except one new occurrence of a dog guarding a rawhide was reported in the third month. For dog identified with food guarding, the return rate to the shelter was 5% and 9% for adult dogs not identified with guarding behavior. Adopters did not comply with at least one aspect of the program, so it is unclear why so little guarding was reported. The key finding is that dogs that guarded their food bowl in the shelter were not guarding their food in their new homes.

## 1. Introduction

Dogs have evolved complex mechanisms that help them obtain food and contend with competition [1]. Early domesticated dogs scavenged on what they could find [2] and competition for resources was influenced by genetics, the history of the individual, and amount or variety of food available [3]. These ritualized guarding behaviors have persisted in dogs, despite domestication [4]. Canids may guard by lip-curling, making direct eye contact, stiffening their body, using an array of vocal behavior and even biting when approached [5]. Other resources that may be guarded are mates, offspring, sleeping sites [1], proximity to favorite spaces, and proximity to people [6]. Guarding behavior is ritualistic and is not designed to be injurious [4]; however, it poses a potential danger to people who do not respond appropriately to canine body language. 

Dogs may guard resources both in people’s homes and also when housed in animal shelters where resources may be even more scarce. Many animals will display a higher level of aggression when the resource is scarce [7]. This research shows that animal shelters frequently encounter food bowl guarding and this behavior often decreases a dog’s adoptability. Although some shelters will adopt dogs that exhibit these behaviors, many place rigorous adoption restrictions that severely limit placement options. Moreover, in an effort to meet their ethical duty to place safe dogs into the community, many shelters across the country are euthanizing young and healthy dogs that would be highly adoptable if not for their food guarding behavior.

This research focuses on food guarding from people, which is limited to the specific guarding behaviors around the food bowl only when a person is near. The objectives of this study were to (1) identify adult dogs that exhibited food bowl guarding behavior in the shelter, (2) place dogs into adoption without structured training sessions around the food bowl, (3) evaluate occurrence of all guarding behavior in the home for 3 months, and (4) quantify how this behavior was being addressed in other shelters across the country. These results can be used to develop guidelines to enable shelters around the country to save more lives and place more dogs into adoption.

## 2. Materials and Methods

### 2.1. Study Site

This study was performed at the Wisconsin Humane Society (WHS) in Milwaukee, Wisconsin, USA from April 2004 to September 2006. This was the only humane society in Milwaukee County. WHS was a limited-admission shelter with an intake of 15,000 animals per year. Their animal population consisted of animals surrendered by their owners and animals transported from other shelters and animal control. 

### 2.2. Assessment

During this time, there were 6,603 adult dogs placed into adoption and of those, 96 dogs were placed into adoption on a food guarding program. All dogs were housed individually. Food bowl guarding was determined by the ASPCA’s Safety Assessment For Evaluating Rehoming (SAFER) aggression assessment in dogs greater than 6 months of age [8]. This seven-item assessment was developed by one of the authors (EW) and is used nationwide. The assessment focuses on identifying aggression in a variety of contexts, including guarding of the food bowl and toys. All assessments were performed between 2–3 days after intake and all were videotaped. Each item of the assessment resulted in a score between 1–5. Dogs that scored a “1” or “2” were dogs that exhibited non-aggressive highly adoptable behavior and require little to no resources for adoption. Dog that scored a “3” exhibited stiffer body language and dogs that scored a “4” or “5” were those who growled and/or attempted to bite the handler and need greater resources. The observable objective behaviors for each score, item by item, can be seen on the ASPCA Pro website [9].

### 2.3. Detection of Food Aggression

The food assessment item consisted of placing dry dog food into a bowl with a few tablespoons of canned dog food and placing the bowl on the floor. Once the dog begins eating, a fake hand was used to pull the bowl one foot (30 cm) towards the assessor. A second approach was made to guide the dog’s head out of the bowl with the fake hand on each side of the dog’s head [9]. If the dog did not eat, another type of canned food was offered in order to get an eating response before taking the bowl away. If they still didn’t eat, they were put back into the kennel and the food item was repeated later in the day.

### 2.4. Criteria for Food Program

Inclusion criterion were dogs over 6 months of age who scored a “1” or a “2” on all items of the assessment and they scored a “3, 4 or 5” on the food bowl item. The goal was to have aggressive behavior only pertaining to the food bowl being taken away and not in any other circumstances. Guarding behavior could be mild to extreme and included stiffening, gulping, growling, freezing, and/or biting the fake hand while eating. 

Exclusions from the program were dogs that were less than 6 months of age and pit bulls or Rottweilers (as required by the shelter). This was a preliminary study and dogs showing the following behaviors were excluded for safety reasons: left the bowl more than 12 inches (30 cm) to bite the incoming fake hand, advanced up the hand while biting multiple times, picked up and moved the bowl with their teeth, placed their body between the assessor and the food bowl, placed a foot into the bowl while guarding, and urinated on the bowl. The in-home protocol only addressed behaviors around the food bowl, so any dogs that also guarded toys or other non-food items were excluded from this study.

### 2.5. Protocol Pre-Adoption While in Shelter

After the assessment, all dogs were placed on a free feeding program at the shelter, with dry kibble available 24 hours a day in the dog’s kennel. All dogs were given daily foraging enrichment in their kennel such as placing their kibble into an enrichment device and daily physical exercise in the form of walks or playtime in a yard with a person and/or another dog. Dogs were moved into adoption within a few days and a sign identified them as being on the food program. Dogs were not re-assessed for guarding behavior before going home because taking the bowl away was counterproductive to the food program and it could reinforce the guarding behavior.

### 2.6. Adoption

Upon adoption, all adopters signed a waiver acknowledging the dog’s food aggression as well as agreeing to follow an in-home food guarding protocol. There were no controls for this study as all food guarding dogs were adopted and given the same follow-up support and protocol for safety and humane reasons.

### 2.7. Protocol Post-Adoption in-Home

Each adopter was instructed to follow a “food program” that consisted of the following: food available at all times; feed a portion of the dog’s daily ration out of enrichment or foraging device; avoid excitement about feeding time; and have the dog sit before lowering the food bowl to the floor. Adopters were to avoid conflict with the dog during mealtime (e.g., placing their hands into the bowl or repeatedly taking the bowl away). They were advised to allow the dog to eat without disturbance. The only interaction around the food bowl was structured and based on positive reinforcement by dropping a high value treat into the bowl while they walked past the bowl. Within a week after adoption the adopter returned to the shelter with their dog for a private consultation to ensure the adopters understood the protocol and to address questions. The in-home protocol is used by the ASPCA^®^ and can be found on Table 1. 

### 2.8. Follow-Up

A follow-up telephone call was given post-adoption at 3 days, 3 weeks, and 3 months. Up to three phone attempts were made at each follow-up by a key person in the behavior department. A scripted questionnaire was used on each call and included open ended questions about type of food being fed, behaviors in the home, general interactions with the dog, and if there had been any major changes in the dog’s life or their schedule since adoption. Also asked were yes/no format questions regarding free feeding, enrichment offered, if they could pick up the dog’s food bowl or toys, and if the dog had been returned or they were planning on returning the dog. Then, on a 1–10 scale with 10 being most attached, adopters were asked to rate their level of attachment to the dog and how attached they thought the dog was to them.

**Table 1:** The food modification program that adopters were instructed to follow after adoption of a dog identified with food guarding.

As your adoption counselor discussed with you, your dog displayed food guarding while in the shelter. This means that your dog may be more likely to show aggression around his food than some other dogs. Food-aggressive dogs may bite when they perceive that someone is trying to take their food. Food aggression is both manageable and controllable. We highly recommend you follow the plan below, beginning the moment your dog enters into your home.
The Plan:
**Food time should never be made into an event.** Do not get the dog riled up for dinner. **Be sure your dog sits and waits for the food bowl.** For the first few days, you might want to keep the leash on the dog for this exercise. As the dog sits, you will bend toward the dog with the bowl. If the dog gets up, stand up and have the dog sit again. Repeat until he can stay seated while you lower the bowl. **Put small amounts of food in the bowl.** As the dog finishes the first few bites, place more food in the bowl. Feed the normal amount of food, but do so in small amounts. **Feed one-half of the dog’s food out of a food-dispensing toy such as the Buster Cube.™** This will not only help with food issues, but it will also help keep your dog busy both in body and mind. **When your dog is interacting with the food-dispensing toy or eating food from the bowl, you can teach him that when he leaves the bowl or toy to look at you, he will get something even better.** This is a very important and fun exercise! Begin by placing dry kibble in the bowl or food-dispensing toy. Let the dog eat for a moment, then walk over with a tasty piece of cheese or other highly desirable food item. Say the dog’s name. If he lifts his head, praise him and give him the food item. **Trade.** While this game is similar to what is written above, here you are not only asking the dog to lift his head, but to let you have one object for another more desirable one. Place a tasty treat that the dog loves in your pocket, and begin by giving the dog a boring toy — one that the dog finds only mildly interesting. We want the dog to quickly understand the game as well as avoid any aggression, so we must begin by giving the dog something that is not highly desirable.) Once the dog has this item for a moment, take the tasty treat out of your pocket and calmly say “Trade.” Draw the dog toward you with the treat, and let him nibble the treat while you pick up the boring toy. When he is finished with the treat, have him sit and return the toy to him. We want your dog to learn that you always have something better and that he can trust you.
If you are unable to do the preceding exercises, we suggest you choose another dog to adopt. We want you to be safe and for the dog to have the opportunity to be able to work through his issues. Please take the time to ask yourself if you are ready to take on a dog that will require more time and resources than some other dogs we have available for adoption.

### 2.9. Subset Study: Shelter Survey on Food Guarding Programs

To complement research at WHS, the ASPCA^®^ developed a short survey to quantify guarding behavior in shelters around the country and to learn more about what programs were being used. This survey was sent once via email to 350 shelters nationwide using Survey Monkey. Participants were obtained from various Yahoo list serves and the ASPCA^®^ email contact list. Seventy-seven separate organizations responded to this voluntary survey from 28 September to 5 October 2006. Duplicate entries from the email or the computer were not allowed by the software. The survey included many open-ended questions such as: identifying the source of the dogs in their shelter, what behavioral assessment was used (if any), and the top three behavioral reasons a dog was found to be a non-adoption candidate. Also asked was the number of dogs identified with food guarding and other types of aggression, and the outcomes of those dogs. It was recommended that the survey be completed by someone knowledgeable in the evaluation process in order to report accurate numbers and provide enough detail to answer all the questions.

## 3. Results

### 3.1. Source

Dogs in this food guarding program came from these intake sources: 41% were owner-surrender to the facility, 31% were transferred from the nearby animal control, and 28% were transfers from other shelters.

### 3.2. Food Guarding Behaviors Reported

Follow-up calls were made at 3 days, 3 weeks, and 3 months with a 69% response rate for at least one follow-up. Of the dogs that were adopted, 30 adopters (31%) were never reached for follow-up calls. Of those, 24 never returned messages on their voicemail, three people did not have voicemails, two people left a number that was disconnected, and one was the wrong number. A total of six dogs were returned over three months. Of the remaining 60 adopters with follow-up calls, 17% were reached only once, 32% were reached twice, and 52% were reached during all three follow-up calls. Of all owners reached, 84% of them had two or more follow-up calls (Table 2). 

At the first follow-up on the third day, three adopters reported some type of guarding behavior (Table 3). Dog 1 growled when a rawhide was taken away, Dog 2 guarded the food bowl and toys (she was returned), and Dog 3 growled when a tennis ball or a rawhide was taken away. At the second follow-up, 3 weeks after adoption, Dogs 1 and 3 remained in their home and continued to exhibit the same guarding behaviors and two other dogs (Dogs 4 and 5) had a guarding event over a specific chew toy. The adopter of Dog 4 was not reached in the first follow-up so it is unclear when this behavior first appeared. At the 3 month follow-up, adopter’s reported that Dogs 1, 3, and 5 no longer exhibited guarding behaviors and the adopter of Dog 4 was not reachable. One new report of guarding was noted in Dog 6 who growled when a rawhide was taken away but not the food bowl. 

**Table 2:** All dogs that had at least one response to a follow-up call post-adoption (n = 60). Dogs are organized by number of times they were reached over all three attempts and do not include the six dogs who were returned.

	Dogs with at least one follow-up	3 day	3 week	3 month	Total number of times reached
		n = 52	n = 54	n = 35	
**1**	Bono	1	0	0	1
**2**	Cherie	1	0	0	1
**3**	Daphne	0	1	0	1
**4**	Freckles	0	1	0	1
**5**	Fred	0	1	0	1
**6**	Hershey	0	1	0	1
**7**	Hunter	1	0	0	1
**8**	Lucky	0	1	0	1
**9**	Rufus	0	1	0	1
**10**	Ziggy	1	0	0	1
					10 (17%)
**11**	Achilles	1	1	0	2
**12**	Bailey	1	1	0	2
**13**	Benny	1	1	0	2
**14**	Bogey	1	1	0	2
**15**	Cody	1	1	0	2
**16**	Daisha	1	1	0	2
**17**	George	1	1	0	2
**18**	Gracie	1	1	0	2
**19**	Jack	1	1	0	2
**20**	Jamie	1	1	0	2
**21**	Lily	1	1	0	2
**22**	Maggie	1	1	0	2
**23**	Nate	0	1	1	2
**24**	Pierre	1	1	0	2
**25**	Sarah	1	0	1	2
**26**	Sasha	1	0	1	2
**27**	Squirt	0	1	1	2
**28**	Sunny	1	1	0	2
**29**	Zoe	1	1	0	2
					19 (32%)
**30**	Amanda	1	1	1	3
**31**	Apple	1	1	1	3
**32**	Bowser	1	1	1	3
**33**	Brownie	1	1	1	3
**34**	Butch	1	1	1	3
**35**	Buttercup	1	1	1	3
**36**	Daisy	1	1	1	3
**37**	Farfel	1	1	1	3
**38**	Ferris	1	1	1	3
**39**	Finn	1	1	1	3
**40**	Fritz	1	1	1	3
**41**	Hercules	1	1	1	3
**42**	Indy	1	1	1	3
**43**	Jimmy	1	1	1	3
**44**	Lola	1	1	1	3
**45**	Lyle	1	1	1	3
**46**	Mattie	1	1	1	3
**47**	Maya	1	1	1	3
**48**	Milo	1	1	1	3
**49**	Nala	1	1	1	3
**50**	Nina	1	1	1	3
**51**	Petunia	1	1	1	3
**52**	Piper	1	1	1	3
**53**	Poncho	1	1	1	3
**54**	Rex	1	1	1	3
**55**	Scruffy	1	1	1	3
**56**	Sophie	1	1	1	3
**57**	Sparky	1	1	1	3
**58**	Spencer	1	1	1	3
**59**	Tasha	1	1	1	3
**60**	Twix	1	1	1	3
					31 (52%)

Four other dogs were included in Table 3. Dog 7 was reported to always eat rapidly but the owner did not report any guarding behavior of food or non-food despite picking up the bowl while the dog was eating, not offering enrichment, and not free feeding. Dog 8 was reported to be “a little bit nervous with toys” but no guarding behavior of food or non-food was reported. Dog 9 was returned due to mouthing with pressure to arms, legs, and clothing to all family members, including the children. The children were often left unattended with the dog and had bitten and left bruising on several occasions. The adopters reported that the dog was not guarding the food bowl and they could pick up the food bowl while the dog was eating and take toys away. Dog 10 was also returned as he had bitten the owner and child each once “when the dog was being ignored” and also bit a passerby while out on a walk. The bites did not break skin and the owner reported no guarding in the home despite picking up the food bowl while the dog was eating and taking toys away from the dog.

**Table 3:** All dogs that were reported to have any concerning behavior in-home during follow-up (both guarding and non-guarding behaviors are included).

Guarding behavior reported	Dog	3 day	3 week	3 month
Yes	Dog 1	Growl when rawhide taken	Growl when rawhide taken	No guarding
Yes	Dog 2	Guards food, toy, & objects: returned	n/a	n/a
Yes	Dog 3	Growl when a tennis ball or rawhide taken	Growl when a tennis ball taken	No guarding
Yes	Dog 4	Unable to reach adopter	Growl when plastic bone was taken	Unable to reach adopter
Yes	Dog 5	No guarding	Growled once over a rubber toy	Some separation anxiety: no guarding
Yes	Dog 6	No guarding	No guarding	Growl when rawhide taken away
No	Dog 7	Eats food fast but no guarding	Eats food fast but no guarding	Eats food fast but no guarding
No	Dog 8	“Nervous” with toy, no guarding with food	No guarding	No guarding
No	Dog 9	Mouthy; bit & broke skin	n/a	n/a
No	Dog 10	Bit a passerby on leg while on a walk.	n/a	n/a

### 3.3. Interactions in the Home

Adopters did not comply with all aspects of the in-home program over the 3 month period (Table 4). About a third of the adopters reported that they provided their dogs with an enrichment feeding device daily. Two-thirds of adopters reported that they consistently asked the dog to sit before lowering the food bowl. Adopters were instructed to let the dog eat and not disturb the bowl while eating, yet during the first 3 days, over half reported that they had picked up the dogs’ bowl while eating and most reported they had done this by 3 months. The majority of adopters reported taking toys out of their dog’s mouths in the first few days of being home (there were no restrictions on this in the protocol) with almost everyone having done so by 3 months. Only 32% of adopters were free feeding their dogs during the first 3 weeks, and 58% were doing so by 3 months. In the six homes where guarding occurred, those adopters were just as inconsistent in following the protocol as adopters who did not observe guarding, although they did share some characteristics. They all attempted to pick-up the bowl while the dog was eating, all took toys away while the dog was playing, all responded yes to having a major life change, and only one was free-feeding while the rest fed at mealtimes. 

**Table 4:** Adopters’ responses at each follow-up to questions pertaining to interactions between them and their dog.

Responses on follow-up calls		Are you using enrichment device to feed part of the daily ration?	Are you asking the dog to sit before putting down the food bowl?	Can you pick up the food bowl while he’s eating?	Can you take toys away?	Do you leave food available all day?
3 days n = 52	Yes	42%	66%	59%	73%	32%
	No	58%	32%	7%	2%	61%
	Unknown	0%	2%	34%	25%	0%
3 weeks n = 54	Yes	37%	68%	82%	92%	32%
	No	63%	32%	5%	2%	68%
	Unknown	n/a	0%	13%	6%	n/a
3 months n = 35	Yes	39%	58%	89%	97%	58%
	No	58%	42%	3%	0%	42%
	Unknown	3%	0%	8%	3%	0%

Two additional questions were asked at 3 months. The first was, ”Do you feel that your knowledge about food guarding with [dog’s name] has changed the way you have behaved around him/her?”, to which 58% of adopters answered “yes” and 42% responded “no”. Many commented that they were (1) taking more precautions around the dog than they would have otherwise and (2) implementing some of the protocols in order to prevent food guarding in the home. The second question asked was “Have there been any other significant life changes since adopting?” to which 37% said yes (63% said no). Some of those life changes were working longer hours, taking long trips, moving, and loss of another pet.

### 3.4. Returns

The return rate for all adult dogs at WHS between 2004 and 2005 was 9%. Of the 96 dogs in the food program, five dogs (5%) were returned within the first week. Reasons for return were (1) two dogs showed aggression not related to guarding, (2) one dog was too active and (3) two adopters were not ready for a dog. Three weeks after adoption, one more dog was returned for separation anxiety. No additional returns occurred during the duration of the 3 month follow-up. 

### 3.5. Attachment

When asked how attached they were to their dog, the majority of adopters gave high rating at all follow-up calls (Table 5). When asked how attached their dog was to them, the majority of respondents gave the high rating at all follow-up calls with the highest score occurring at 3 months. 

**Table 5:** Responses to the two questions “On a scale of 1–10, with 10 being the most attached: how attached are you to your dog?” and “how attached is the dog to you?

	Adopter’s attachment to dog			Adopter’s perception of their dogs attachment to them		
	3 days	3 weeks	3 months	3 days	3 weeks	3 months
	n = 52	n = 54	n = 35	n = 52	n = 54	n = 35
Unknown	2%	0%	0%	3%	0%	0%
1	0%	0%	0%	0%	0%	0%
2	0%	0%	0%	0%	0%	0%
3	0%	0%	0%	0%	0%	0%
4	0%	0%	0%	0%	0%	0%
5	2%	0%	0%	2%	0%	0%
6	2%	0%	0%	2%	0%	0%
7	0%	0%	3%	5%	3%	0%
8	9%	10%	0%	3%	7%	0%
9	20%	9%	16%	9%	8%	11%
10	66%	81%	82%	76%	82%	90%

### 3.6. Subset Research: Shelter Survey on Food Guarding Programs

There were 77 shelters that completed the ASPCA^®^ survey that spanned across 26 states. Eighty one percent of those shelters accepted strays, 99% took owner relinquished animals and 37% served as animal control for their community. A majority (76%) transferred animals into their facility and 66% transferred animals to other facilities. Most (89%) used an assessment on dogs before adoption (Table 6). 

**Table 6:** Responses to the open-ended question: “Please describe the assessment that your shelter uses on dogs”.

Type of Assessment	Percentage who used this assessment n = 77
ASPCA® SAFER™	19%
Sue Sternberg Assess-a-Pet	15%
A blend of SAFER™ & Assess-a-Pet	27%
One used internally that they developed themselves	12%
Did not report an assessment	27%

The top 3 reasons a dog was identified as unadoptable were: aggression while guarding food or non-food items, aggression to an adult or child, and aggression to other dogs (Table 7). Not all respondents provided an answer to this question.

**Table 7:** Responses to the open-ended question: “Name the top three behavioral reasons dogs are identified as unadoptable at your facility”.

Behavior reported	Total number of responses n = 59
Any guarding behavior	42
Aggression to people	41
Aggression to other dogs	22
Aggression to animals	18
Fear/anxiety	15
History prohibits adoption	5
Too stressed in kennel	5
Lack of social behavior	3
Over-arousal	2
Escape behavior	1
Separation Anxiety	1
Too old/aging problems	1
Unpredictable behavior	1

Despite the variety of assessments used, 92% assessed for food guarding behavior. On average, shelters reported that 14% of their dog population exhibited food guarding behaviors during an assessment. The range was 7% to 30% with one shelter reporting 2,000 dogs per year showing food guarding. If a dog guarded the food bowl, only 34% of the shelters attempted to modify the behavior and 51% said they made no attempt to offer dogs for adoption if they exhibited food guarding behavior of any kind. Half of the respondents (52%) reported they had some resources for behavior modification which included using trainers or behaviorists, enrichment programs, hand feeding, volunteer-run training programs both in-shelter and foster homes, and sending dogs through a group training class on-site. Based on their own post-adoption follow-up, shelters in the survey reported that food guarding was not seen in the home presumably because adopters were managing the environment around the dog and not placing them into situations where they would likely guard. 

## 4. Discussion

This study identified dogs with food bowl guarding from one large shelter and included males and females, owner surrender and strays. The only behavior modification performed in the shelter before adoption was ensuring all dogs were free fed and they had daily foraging enrichment opportunities. In the 3 month follow-up post adoption, this study found that people became highly attached to their dog within 3 days; however, they did not follow many of the recommendations given to them at adoption. Despite that, six adopters reported guarding of non-food items in the first three weeks and of those, only one dog guarded the food bowl. By 3 months those dogs no longer exhibited any guarding behavior and one new occurrence of guarding appeared at 3 months over a rawhide. The return rate was lower than the dogs not in this study group.

Some research suggests that a large component of reducing food guarding is making food readily available. Scott and Fuller noted when puppies housed as a litter were free fed; guarding was rarely observed [10]. When resources are plentiful there is less aggression while aggression increases with competition [1]. When food guarding is identified in a shelter dog, they should have food available at all times and provided multiple bowls when sharing a kennel to ensure adequate food availability and low competition. Free feeding in general may help reduce the likelihood of food guarding behaviors during assessments. Future research could include free feeding those dogs who food guard while housed at the shelter, then possibly re-assessed before adoption to measure the impact of free feeding on guarding behavior. If free feeding alone could reduce guarding behavior, than this could be a safety net for many dogs in low resource shelters. It is important to note that of the six dogs found guarding post-adoption in this study, only one adopter was free feeding and all were attempting to pick-up the bowl while the dogs were eating. Given that aggression is a normal component of the dog’s behavior [11] and the ease with which dogs learn to guard, the probability of guarding in the home could be decreased with more post-adoption support (such as private session in the home from a skilled dog trainer or animal behaviorist). 

Odor is an important component of food selection for dogs [12] and many canids will increase guarding behavior when the resource has stronger odor and therefore, higher value [13]. During the ASPCA SAFER™ assessment, dogs are given an un-basted basted rawhide and not given items with strong odor or other types of chew items. The assessment was designed to capture guarding behavior around something of value to a dog that can be found in any home, such as food in a bowl. This assessment does not give pig ears and other types of food-based chew items that have a strong odor. Chews items available to an adopter come in a wide array of texture, odor and shape so it would be difficult to assess how a dog responds to all of them. The shelter could consider setting restrictions for adopters for those higher-value treat items during the first few weeks since most of the guarding behavior observed was towards these items. It is possible that food guarding dogs may be more likely to guard high-value treats in the first three weeks post-adoption. It is just as likely that any dog would also guard those same treat items when they first arrived in a new environment. Further investigation into all dogs post-adoption is warranted. Management of the dog’s environment may be very important in the first few weeks for any newly adopted dog, and especially if seen guarding their food during an assessment. 

Vocal and other guarding behavior also does not mean biting is certain. Canids have developed ritualized signals to gain resources without injury [14]. Often dogs will use vocal behavior such as growling, snapping, barking, and snarling, and even though these sounds make people feel uncomfortable, they do not necessarily predict the probability of biting [15]. In one study, over 41% of 3,226 dogs showed aggressive vocal behavior towards people [16]. Of those, only 15% ever bit a human and less than 10% of those bites caused injury. Bradley reports that this bite percentage is likely lower than reported, as many respondents qualified “mouthing behavior” as biting. The only real predictors of biting are in the situations of those dogs that have bitten before [3]. Future research could include follow-up on dogs that vocalized during food guarding and compare those that bit the fake hand to those that did not to see if there is a difference in guarding behavior in the home. 

When the 3 week follow-up was compared to the 3 month, people offered enrichment less often, fewer asked their dog to sit before lowering the food bowl, and more people picked up the bowl while their dog was eating, more left food out all day, and more people took toys out of their dog’s mouth. It is difficult to know which one of these variables had a greater effect behavior. Despite some lack of compliance by all owners, reports of guarding were low and only one dog guarded the food bowl. Adopters may not be compliant with the protocol, but many reported they behaved more cautiously due to receiving basic education about body language and guarding. Due to inconsistency with adopter compliance, it is not clear what aspects are most useful, and the authors cannot rule out that food bowl guarding may decrease by simply placing the dog into a home. 

As time passed, fewer adopters responded to calls. However, 84% of those with follow-up had two or more calls, so tracking changes in the individual dogs was quite valuable. Also, if guarding had an equal chance of occurring in all dogs, the non-response bias should have minimal impact on these results. 

There are many possible explanations (beside the food program) for why there is so little guarding in the home when compared to during the assessment. The home environment offers: increased free-feeding and/or enrichment, less competition, generally less stress, greater frequency of daily interactions with people, casual interactions around food, and possible training using food treats. Guarding during an assessment could be an indicator for overall stress levels, lack of enrichment or exercise, and even as a response to the fake hand used to move the food bowl, rather than actual food behavior around the food bowl when people are near. 

Results from the adopters in this research and those reported in the survey suggest that food guarding was managed in the home by the adopters. The recommended in-home guarding program for adopted dogs focused on avoiding the triggers that would elicit aggression (ex: reaching in to take the food bowl away). This is a standard recommendation given by other behavior professionals [17]. Of the adopters who reported guarding behavior, only one guarded the food bowl and the others guarded a specific object such as a rawhide, plastic bone, or a tennis ball. When the dog is guarding a specific item such as these, it is easy for the adopter to manage this by not giving them those objects. 

Further study could evaluate the bond between the adopters and these dogs. The small sample size with both paired and unpaired responses does not allow for significant power of statistical differences. However, the major patterns of attachment are clear. The adopters felt strongly attached to these dogs as early as 3 days and throughout the follow-up, and also felt the dogs were attached to them. A useful comparison would be these same questions given to all adopters who adopted a dog, not just those on the food program. This may also provide more insight to the 4% lower return rate from dogs in the food program. 

Some shelters may perceive certain behaviors, such as food guarding, as a barrier to successful re-homing, but those behaviors may not be a barrier to all adopters. In this research, the adopter chose to adopt a dog that was identified with food guarding behavior but otherwise, showed highly adoptable behavior. The shelter set-up the adopter’s expectations that the dog would guard its’ food and provided behavior support post-adoption. The majority of adopters were highly bonded within the first few days and many used positive words to describe their dog’s personality. Perhaps some shelter restrictions placed on dogs are not actually restrictions for all adopters. Further investigation into adopter’s perceptions and expectations of a shelter dog or specific behaviors should be evaluated. 

We acknowledge that the shelters in our nationwide survey are not a random or representative sample and that we don’t have follow-up information or return rates for those other shelters that placed food guarding dogs. Since there is not a central database that contains statistics about which shelters use an assessment or the outcomes, or even what shelters exist in the US, comparisons cannot be made between the respondents to this survey and shelters who did not respond to the survey. However, dogs included in our study provide valuable preliminary insight into this complex issue. 

This research focused on adopting adult dogs without formal training sessions while in the shelter. However, since this research was completed, the ASPCA^®^ has developed an in-shelter food bowl guarding program [9]. This can be used while the dog is housed in the shelter or while in a foster homes. However, based on our food guarding survey, many shelters do not have the time or skill set to implement food behavior modification before adoption despite 14% of the shelter population exhibiting this behavior. 

More research needs to be done to determine why there is so little guarding observed in the home. The key finding is that they are not guarding. The survey revealed that this behavior does result in euthanasia in shelters around the country and this euthanasia decision may no longer be appropriate. For a large subset of dogs that guard their food bowl, this can be a life-saving program that requires minimal skill and resources for shelters. 

## 5. Conclusions

This study demonstrates that a subset of dogs that guard their food bowl during an assessment can be adopted with infrequent guarding in the home and seldom be returned. Many animal shelters do not have the resources to implement behavior modification for food guarding before adoption and this behavior is a common reason for euthanasia. Adopters did not comply with at least one aspect of the program, so it is unclear why so little guarding was reported. The key finding is that dogs that guarded their food bowl during an assessment were not in their new homes. 
